# Fluorescent CdTe/ZnS Core/Shell Quantum Dots for Sensitive Metabolite Detection in Real Samples

**DOI:** 10.1007/s10895-025-04138-9

**Published:** 2025-01-21

**Authors:** Melahat Sevgül Bakay Ağbulut, Erdem Elibol, Musa Çadırcı, Tuna Demirci

**Affiliations:** 1 Faculty of Engineering, Department of Biomedical Engineering, Duzce University, Duzce, Türkiye; 2 Faculty of Engineering, Department of Electrical and Electronics Engineering, Duzce University, Duzce, Türkiye; 3 Scientific and Technological Researches Application and Research Center, Duzce University, Duzce, Türkiye; 4 Nanotechnology Research Laboratory, Duzce University, Duzce, Türkiye

**Keywords:** CdTe/ZnS QDs, Fluorescence detection, Vitamin C, Glucose, Folic acid

## Abstract

This study highlights the aqueous synthesis of CdTe/ZnS core/shell quantum dots (QDs) and their application as fluorescence sensors for detecting critical metabolites, including folic acid, glucose, and vitamin C, in real biological samples. The synthesized QDs exhibit excellent quantum efficiency, stability, and biocompatibility, enhanced by mercaptopropionic acid (MPA) ligands, enabling eco-friendly and accurate sensing. Detection limits of 0.84 µg/mL for folic acid, 0.33 mM for glucose, and 1.15 µg/mL for vitamin C were achieved with high linearity (R^2^ > 0.97). These results underscore the potential of CdTe/ZnS QDs in advanced biosensing technologies, offering sensitive and selective metabolite detection through a robust FRET-based mechanism. The versatility and aqueous solubility of these QDs pave the way for their integration into multiplex diagnostic systems for enhanced biomedical applications.

## Introduction

In recent years, semiconductor nanocrystals which are known as quantum dots (QDs) have garnered a great deal of attention due to their unique size-dependent features that set them apart from their bulk counterparts. The change in diameter of QDs in the range of 1–10 nm provides specific and unique optical and electrical properties. These distinctive features also enable QDs to use in different application areas especially solar cells [1–4], light-emitting diodes [5–7], biomedical imaging [8–10], and biological metabolite detection [11–13]. The most direct effect of QD size on metabolite detection while performing biological metabolite detection is its effect on the emission wavelength. The smaller QDs emit light at shorter wavelengths (more blue) and the larger QDs emit light at longer wavelengths (more red). This size-dependent tunability allows researchers to select QDs that emit at specific wavelengths and optimise metabolite detection based on the spectral characteristics of the sample and detection metabolites [14]. The larger QDs tend to be brighter than the smaller ones because they contain more atoms, leading to a greater number of electron–hole pairs available for recombination and light emission. This increased brightness can increase the sensitivity of metabolite detection, especially in complex biological matrices where background noise can be especially of high [15].

Detection and quantification of small organic or inorganic molecules which are called metabolites such as vitamins, fats, hormones, carbohydrates and amino acids from tissues, cells or body fluids in biological systems, play a very important role in disease diagnosis [16]. As a micronutrient, humans require Vitamin C (Vit C) which is also known as ascorbic acid due to the pleiotropic qualities connected to its ability to donate electrons. It is a potent antioxidant as well as a cofactor for several enzymes that control gene expression and biosynthesis. Vitamin C enhances immunological defense by supporting a number of innate and adaptive immune system cells functions. By improving the skin's capacity to scavenge oxidants and preserving the epithelial barrier's ability to ward off infections, vitamin C may also offer protection against environmental oxidative stress [17]. Humans are unable to manufacture Vit C, a vital nutrient, because a crucial enzyme in the biosynthesis route has been lost. For these reasons, it is important to determine the amount of Vit C and keep it within the desired value that a healthy person’s blood serum should contain between 0.6 and 2.0 mg/dL of Vit C [18].

One of the vitamins in the B complex group that is vital is folic acid (FA). FA is water soluble and also called folate or vitamin B_9_. While FA can be synthesized by plants and bacteria, it cannot be synthesized by humans or other animals, making it a necessary nutrient. Deficiency in folic acid has been linked to numerous illnesses including neural tube anomalies, megaloblastic anemia, neurological disorders, other congenital deformities, thrombosis and vascular disease [19]. Furthermore, high FA levels might impede the absorption of zinc and vitamin B12, which can result in a variety of disorders [20]. In healthy human blood, an FA level of 3–6 ng/mL is regarded as normal [21].

Glucose (Glu) is indeed a pivotal molecule in biological systems, playing essential roles in both photosynthesis and cellular respiration. Its significance stems from its ability to serve as a primary source of energy in various metabolic pathways. Additionally, glucose serves as a precursor for the synthesis of vital molecules like amino acids, nucleotides, and lipids. Its central role in metabolism underscores its indispensability for the functioning of organisms across diverse biological contexts [22]. In adults, a standard range for the concentration of glucose in bodily fluids is recognized as falling within the interval of 70 to 100 mg/dL [23].

In general, it is apparent that the monitoring of Vit C, FA, and Glu levels is important for the diagnosis and management of diseases [24, 25]. For quantify these metabolites, different procedures are used such as high-performance liquid chromatography [18, 26, 27], colorimetric [28], electrochemiluminescence [29–31] and electrochemistry [32, 33]. However these methods stand out with their negative aspects such as low selectivity, difficult pretreatment, wasting sample and long analysis time [34]. Comparatively, the traditional methods used to detect these analytes (such as colorimetric assays, electrochemical sensors and enzyme-linked immunosorbent assays (ELISAs)) each have their own strengths and weaknesses. Colourimetric analyses are simple and cost-effective but often lack the sensitivity and specificity of QD-based methods. Electrochemical sensors can offer high sensitivity and real-time monitoring capabilities but can suffer from electrode fouling and stability issues over time. ELISAs are highly sensitive and specific, but can be time-consuming and expensive and often do not support real-time monitoring. Although the sources provided do not provide specific comparisons between QD-based sensing for folic acid, vitamin C and glucose and these other methods, the general advantages of QDs suggest that QD-based sensors can offer competitive sensitivity in many cases. However, the actual sensitivity achieved will largely depend on the characteristics of the sensor design and experimental conditions [35]. Hence, there is a significant need for the advancement of detection technologies that are more precise, selective, cost-effective, and user-friendly. In this regard, the fluorescence-based method for metabolite detection [36] emerges as particularly promising, given its benefits including high sensitivity, ease of use, and rapid response [34] compared to conventional techniques.

In the literature review, it was observed that studies were carried out using CdTe/ZnS QDs synthesized with glutathione ligand for the detection of FA or Glu [37, 38]**.** Studies have emphasized that CdTe/ZnS QDs can be used for metabolite detection by modifying them with Mn^+**2**^**.** CdTe/ZnS QD development increases the efficiency, stability, and biocompatibility of the ZnS shell, resulting in increased photoluminescence and resistance to environmental degradation, both of which are critical for long-term applications [39]. In addition, the study improves detection capabilities in real biological material and FRET-based quenching mechanisms. In previous work by our group for metabolite detection, CdSeTe QD-based research had limitations, especially in terms of quantum efficiency, stability, and biocompatibility, which necessitated these improvements [40]. Although CdSeTe QDs have provided preliminary insights into metabolite identification, their effectiveness in real biological material has unfortunately been limited by fluorescence quenching and poor long-term stability. In contrast, CdTe/ZnS QDs alleviate these issues by adding a ZnS shell that improves optical properties and increases resistance to environmental degradation, resulting in more consistent results for real-world applications [41].

The goal of this study was to perform aqueous synthesis of CdTe/ZnS core/shell QDs with MPA ligand and to investigate the usability of these nanomaterials as fluorescence sensors for metabolite detection in real blood samples. In this context, the synthesis process of CdTe/ZnS QDs for the detection of Vit C, FA and Glu was optimized, the optical and chemical properties of the obtained nanomaterials were characterized and the performance parameters such as sensitivity and selectivity for metabolite detection were tested with standards and blood samples.

## Materials and Methods

### Materials

Cadmium chloride (CdCl_2_, > 99.99%), mercaptopropionic acid (MPA, > 99.0%), tellurium powder (Te, > 99.8%), sodium borohydride (NaBH_4_, > 98.0%), zinc chloride (ZnCl_2_, > 98%), sodium sulfide nonahydrate (Na_2_S.9H_2_O, > 99.99%), ethanol (C_2_H_5_OH, 99.9%), sodium hydroxide (NaOH, > 98.0%), folic acid (> 97.0%) and glucose oxidase (> 95.0%) were supplied from Sigma-Aldrich. D ( +)-Glucose (> 99.0%), hydrochloric acid (HCl, 37.0%) and Vitamin C (injection) were purchased from AFG Bioscience, ISOLAB and local pharmacy, respectively. All chemicals were used as analytical reagents and without any purification. Throughout all experiments, double-distilled water (ddw) was used.

### Characterization

Horiba Fluoromax, Shimadzu UV-1800, Raigaku Smartlab XRD with CuKα radiation of 1.5406 Å diffractometer, Edinburgh Instruments FLS1000 spectrometer, FEI TALOS F200S TEM microscopy operating at 200 kV were used to acquire measurements of fluorescence, UV–Vis, X-ray diffraction (XRD) patterns, PLQY, and HR-TEM, respectively.

### Synthesis of CdTe/ZnS QDs

Water-soluble MPA-capped CdTe/ZnS QDs were synthesized according to previous studies with slight modifications [42]. Firstly, a Cd^2^⁺ precursor solution was prepared using MPA with a molar ratio of 1:2. The solution pH was adjusted to 11 using a 2 M NaOH solution, then transferred into a three-necked flask and mixed under a nitrogen atmosphere at room temperature. For the NaHTe solution, NaBH_4_ and Te were mixed in a molar ratio of 3:1. This solution was initially held under vacuum for 10 min, then exposed to a nitrogen atmosphere, followed by the addition of 10 mL of double-distilled water (ddw). The solution temperature was maintained at 80 °C. After 30 min, the freshly prepared pink-purple NaHTe solution was injected into the Cd precursor solution and stirred at 100 °C for 2 h to synthesize CdTe cores.

Subsequently, a ZnS solution was prepared to form a ZnS shell around the CdTe core quantum dots (QDs). For this purpose, 5 mmol ZnCl₂ and 5 mmol Na₂S·9H₂O were mixed with the addition of 15 mL ddw. To remove oxygen, the solution was stirred under vacuum for 10 min and then under a nitrogen atmosphere for 1 h at 100 °C. The ZnS solution was added dropwise to the CdTe core QDs solution. Following the addition, the mixture was stirred for 2 h at 100 °C, resulting in the formation of CdTe/ZnS core/shell structured QDs. Finally, the QDs were centrifuged at 6000 rpm with ethanol at a volumetric ratio of 1:1, then dried and stored in powder form under ambient conditions for further studies.

### Metabolite Sensing Procedures

Metabolite sensing procedures were conducted on 10 different concentrations for each metabolite. For fulfillment of these processes, Vit C, FA and Glu solutions were prepared in the range of 0.27 µg/mL-8.3 mg/mL, 0.35 pg/mL-0.35 mg/mL and 0.0312 mg/mL-16 mg/mL, respectively. While the first solution called as a blank was formulated by 2.5 mL CdTe/ZnS solution (0.7 µM) and 0.5 mL ddw, other solutions were prepared with (2.5 mL, 0.7 µM) QD solution and 0.5 mL Vit C and FA solutions in varying concentrations. For glucose detection procedures, to realize the enzymatic reaction and release hydrogen peroxide, firstly 0.25 mL glucose solutions in different concentrations and 0.25 mL glucose oxidase (GOx) solutions were mixed for 10 min except for the blank solution. Afterwards, 0.5 mL GOx and Glu mixture were added to 2.5 mL QD solution for fluorescence measurements. All solutions were excited at 410 nm and recorded between 500–800 nm wavelengths.

### Real Sample

The Duzce University Health Application and Research Center provided blood samples from healthy people. The blood was then centrifuged for 10 min at 4000 rpm in order to extract the protein. Vit C, FA, and glucose detection investigations were conducted using blood serum samples and the standard addition method. Before beginning any research, all human subjects gave their informed consent, and all operations followed institutional guidelines as well as any relevant legal requirements.

## Results and Discussion

### Characterization of CdTe/ZnS QDs

**Fig. 1:**
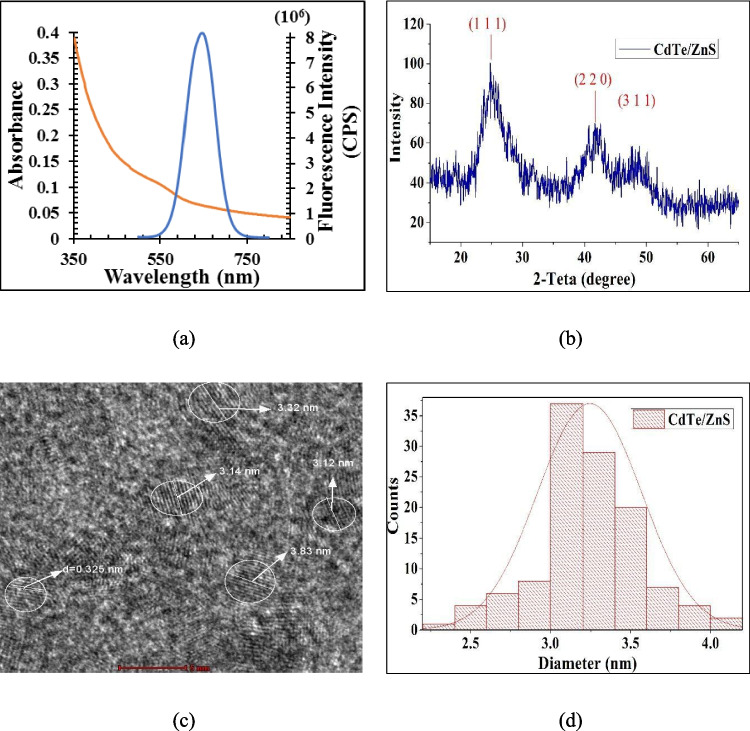
Characterization results of CdTe/ZnS QDs (**a**) UV–Vis and PL (**b**) XRD image (**c**) 5 nm scale HR-TEM image (**d**) size distribution graphic

Following the procedure outlined in the Materials and Methods section, the water-soluble, MPA capped CdTe/ZnS QDs were effectively produced. UV–Vis and fluorescence spectra were employed to investigate CdTe/ZnS QDs’ optical characteristics. The results of the optical property of MPA CdTe/ZnS QDs are displayed in Fig. 1(a), where the QDs demonstrated a broad absorption spectrum with a distinctive peak at 490 nm. When QDs were exposed to 410 nm light, a narrow emission band with a center at 650 nm was seen. The narrow emission spectra suggested that there was a significant level of QD monodispersity. The XRD pattern of the CdTe/ZnS QDs is displayed in Fig. 1(b). The three main peaks for the CdTe/ZnS QDs were found at 24.9°, 41.3°, and 49°. These peaks are related to the zinc blend structure with (111), (220), and (311) planes [42]. QDs’ XRD patterns reveal a highly cubic crystalline structure. Figure 1 (c) and (d) display the interplanar spacing and size distribution of QDs obtaining from HRTEM image. Using transmission electron microscopy, the diameter and interplanar spacing of QDs were determined to be approximately 3.2 nm and 0.325 nm. It can be seen that QDs show high homogeneity and regularity. Consequently, Fig. 1 validates that the synthesized QDs are of high quality and appropriate for application in metabolite detection.

**Fig. 2:**
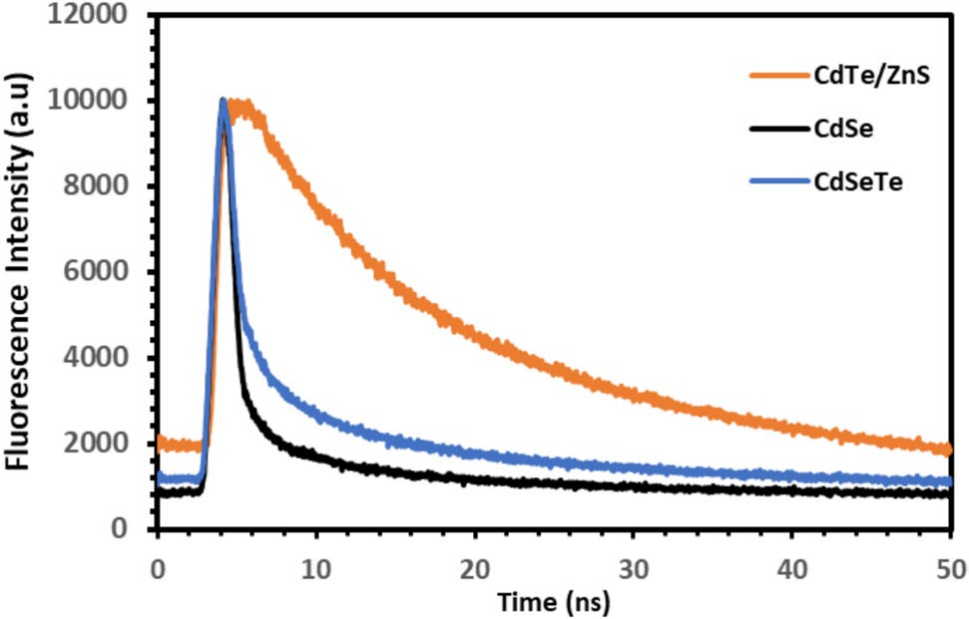
Fluorescence lifetime graphs of QDs

The term 'fluorescence lifetime' is used to describe the average time that QDs remain in the excited state (electron conduction band) before emitting a photon and returning to the ground state (electron valence band). In other words, it defines the time required for the QDs to transition from the excited state to the ground state, i.e. to radiate. This time is typically within the picosecond-nanosecond range [43]. In order to demonstrate the improvement in the fluorescence lifetime of CdTe/ZnS QDs, they were compared with the data of different types of QDs that we synthesized in our previous studies. Excitation was conducted at a wavelength of 377 nm for CdSe QDs, CdSeTe QDs and CdTe/ZnS QDs. The fluorescence lifetimes obtained for QDs are presented in Fig. 2 and in Table 1.

**Table 1:** t_1_, t_2_ times and average fluorescence lifetimes of QDs

	.A1	t_1_ (ns)	A2	t_2_ (ns)	Lifetime (ns)
CdSe QD	6249.78	9.71373	59,758.66	1.76725	2.52
CdSeTe QD	559,503.59	0.9604	3726.06	11.33191	1.03
CdTe/ZnS QD	5995.11	14.26477	5995.11	14.26474	14.27

Furthermore, according to Eq. 1 [44], the average fluorescence lifetimes were calculated as 2.52 ns for CdSe, 1.03 ns for CdSeTe and 14.27 ns for CdTe/ZnS QDs.

1 $$T=\frac{{\sum }Ai\tau i}{{\sum }Ai}$$

A_i_ denotes the amplitudes in the curve equation obtained from the curves fitted to the fluorescence lifetime graphs, τi denotes the fluorescence lifetimes found as a result of the fitted curves.

The data obtained from Table 1 and Fig. 2 show that the fluorescence lifetime of QD is significantly increased by the ZnS shell coating. In fact, the increase in the fluorescence lifetime causes the autofluorescence background to decrease more rapidly and the true fluorescence signal to be distinguished more easily [45]. This allows for more sensitive metabolite detection. It also makes it possible to detect metabolites using lower concentrations of QD [46].

**Fig. 3:**
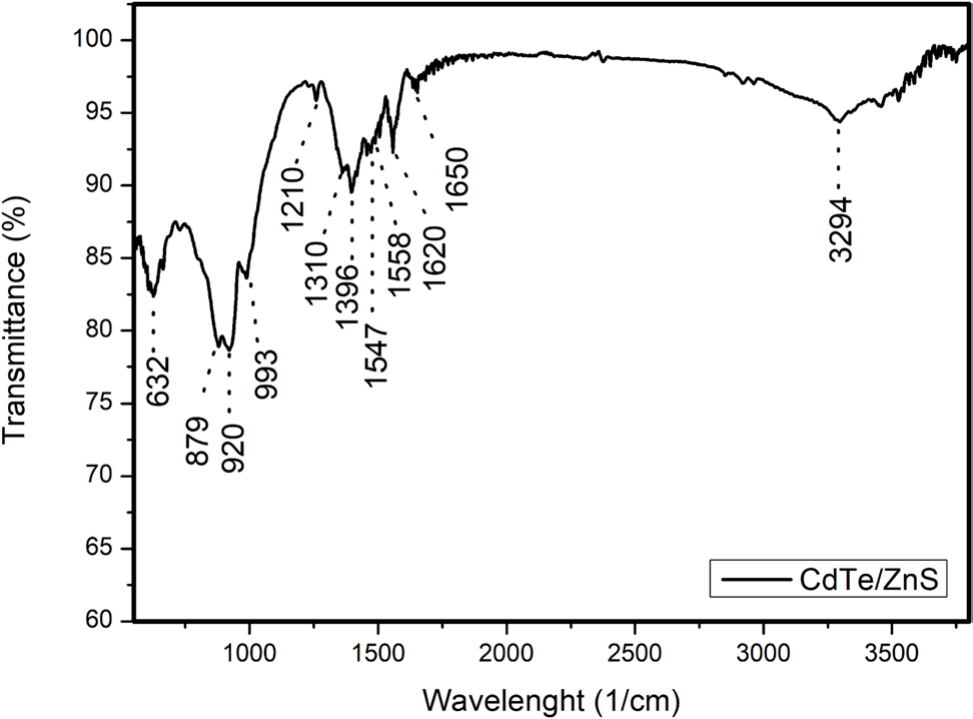
FT-IR graphs of QD

The IR spectrum clearly shows that the broad and strong peaks detected at 3294 cm^−1^ and represent the stretching vibration value of the -OH functional group [47]. The 1650–1620 cm^−1^ range contains vibration peaks formed by C = O bonds in carboxylic acid groups on the surfaces of CNs. These peaks prove that MPA molecules are bound to the surface of CNs. The symmetric and asymmetric vibration peaks formed by -COO- are present between 1547–1558 cm^−1^ and 879 cm^−1^. The -OH bonds arising from carboxylic acid also show vibrational peaks 1396 cm⁻^1^. C-O bonds cause vibrations between 1310 and 1200 cm^−1^. Peaks in the range 993–920 cm^−1^ unquestionably indicate zinc-sulfur bonds in the ZnS shell structure (Fig. 3.)

### Optimization Parameters

In order for metabolite detection measurements to be reliable and effective, key parameters that could affect the fluorescence intensity and optical behavior of QDs were identified and optimized. In this respect, the effects of pH, QD’s concentration and incubation time were investigated for the detection of Vit C, glucose and folic acid by CdTe/ZnS QDs.

**Fig. 4:**
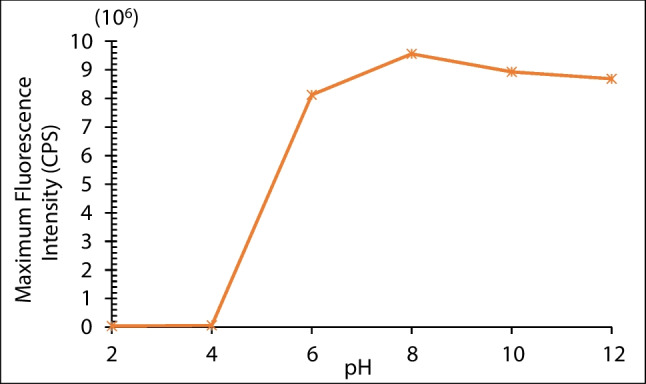
The effect of pH variation on fluorescence intensity of CdTe/ZnS QDs

As a result of measurements made with pH values ​​ranging from 2 to 12, it is clearly seen in Fig. 4 that the acidic environment significantly reduces the fluorescence intensity of QDs. This is probably because the acid (H^+^) may etch the surface S^2−^ to produce HS^−^/H_2_S, which causes surface defects in QDs [48]. The fluorescence intensity is also slightly decreasing after the pH 8, which may be caused by more difficult QDs synthesis or development of defective QDs [47]. Maximum fluorescence intensity is obtained in the pH range of 6.0 and 12.0, thus a pH value of 8 was chosen throughout the study. In the literature, it is likely that around neutral pH (pH 7–8), surface ligands are in a state that optimally passivates the QD surface, minimizing surface defects and maximizing quantum yield. This optimum passivation at pH 8 may be due to the specific properties of the ligands used in the synthesis or functionalization of CdTe/ZnS QDs, such as MPA which may have a pKa value close to this pH range, leading to a balance between protonated and deprotonated states that favors fluorescence enhancement [49]. This demonstrates the agreement of our synthesis with the literature [50, 51].

**Fig. 5:**
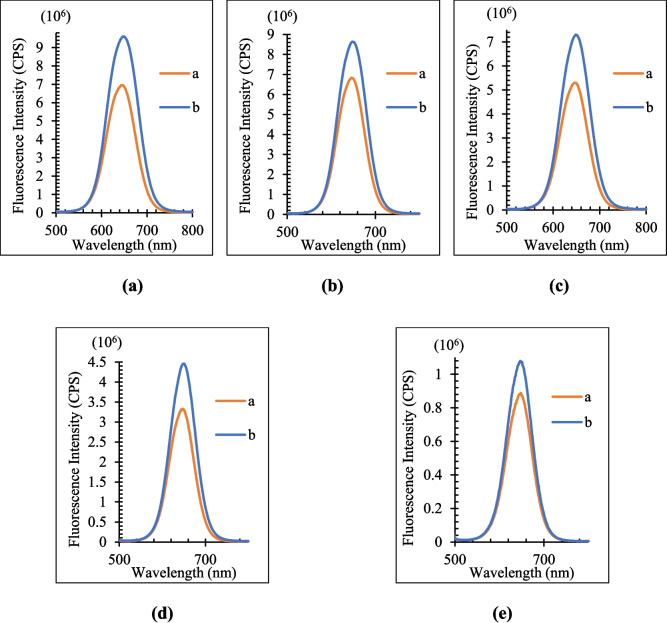
Effects of QD concentration (**a**) 0.7 µM (**b**) 0.56 µM (**c**) 0.42 µM (**d**) 0.28 µM (**e**) 0.14 µM on the fluorescence intensity for Vit C. Orange line represents PL results in the absence of Vit C, blue line represents PL results in the presence of Vit C

**Fig. 6:**
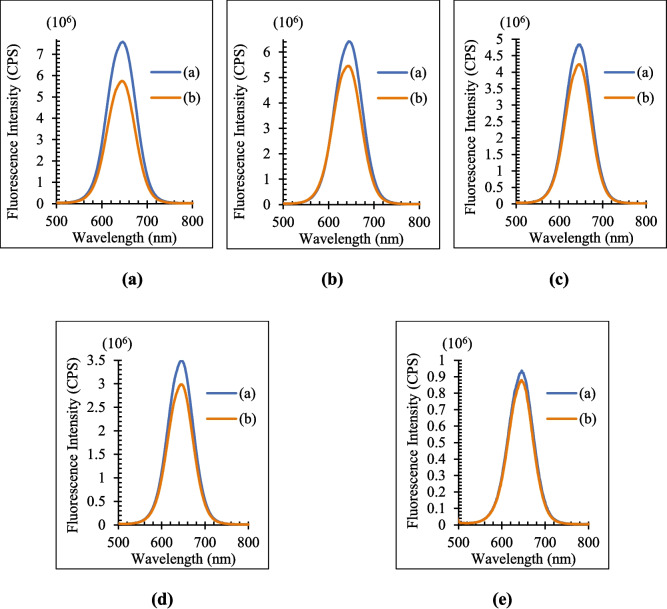
Effects of QD concentration (**a**) 0.7 µM (**b**) 0.56 µM (**c**) 0.42 µM (**d**) 0.28 µM (**e**) 0.14 µM on the fluorescence intensity for FA. Orange line represents PL results in the absence of FA, blue line represents PL results in the presence of FA

**Fig. 7:**
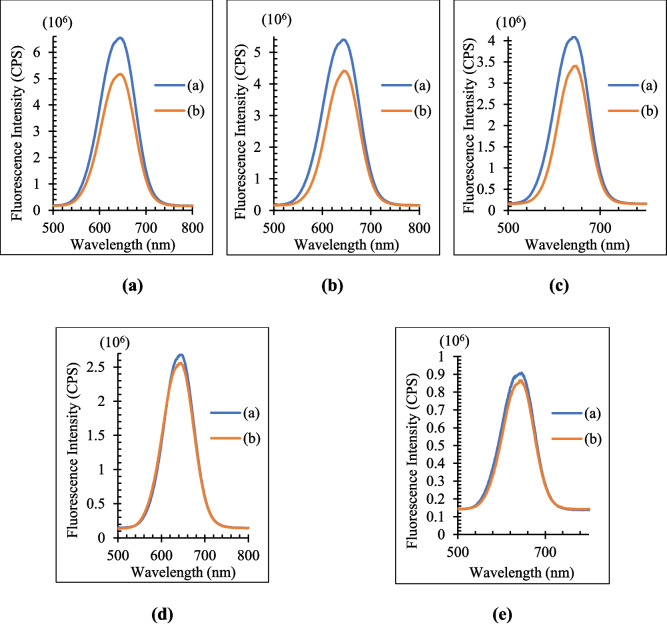
Effects of QD concentration (**a**) 0.7 µM, (**b**) 0.56 µM, (**c**) 0.42 µM, (**d**) 0.28 µM, (**e**) 0.14 µM on the fluorescence intensity for Glu. Orange line represents PL results in the absence of Glu, blue line represents PL results in the presence of Glu

The optimum QD concentration was found by comparing the fluorescence intensity of the blank and metabolite added solutions after five QD solutions with varying molarities were generated for each metabolite. When (I_0_-I)/I_0_ (I_0_: fluorescence intensities of the QD solution in the absence of metabolites, I: fluorescence intensities of the QD solution in the presence of metabolites) is at its maximum, the QD concentration is at its optimal [38]. The fluorescence intensity of blank (b) and metabolite-added (a) solutions for Vit C, FA, and Glu is compared respectively in Figs. 5, 6, and 7. The (I_0_-I)/I_0_ values of each metabolite for the various QD concentrations are likewise displayed in Table 2. The values of (I_0_-I)/I_0_ are highest for all three metabolites at 0.7 µM.

**Table 2:** (I_0_-I)/I_0_ values for metabolites and each QD concentration

		CdTe/ZnS Concentration (µM)				
		0.7	**0.**56	0.42	0.28	0.14
**(I**_**0**_**-I)/I**_**0**_	**Vit C**	**0.382**	0.264	0.378	0.341	0.215
	**FA**	**0.242**	0.150	0.124	0.146	0.062
	**Glu**	**0.210**	0.182	0.169	0.045	0.048

The fluorescence intensity responses of CdTe/ZnS QDs to Vit C, FA, and Glu exhibit distinct trends across varying QD concentrations, underpinned by analyte-specific mechanisms. Fluorescence intensity increases with QD concentration due to the defect-healing effect, where Vit C reduces surface defects, enhancing fluorescence efficiency. This enhancement plateaus at higher concentrations due to defect-healing site saturation and photon re-absorption effects [34]. Fluorescence quenching dominates for FA across all QD concentrations, primarily driven by Förster Resonance Energy Transfer (FRET). The consistent linearity observed in this quenching behavior highlights the strong spectral overlap and proximity-dependent interactions between FA and QDs. Fluorescence quenching for Glu exhibits a linear trend at lower QD concentrations, attributed to a combination of FRET and collisional quenching mechanisms. However, deviations from linearity occur at higher concentrations due to steric hindrance and QD aggregation. These observations emphasize the tunable fluorescence properties of CdTe/ZnS QDs based on analyte type and concentration. Such insights are invaluable for optimizing QD-based sensing platforms tailored for specific biological applications.

**Fig. 8:**
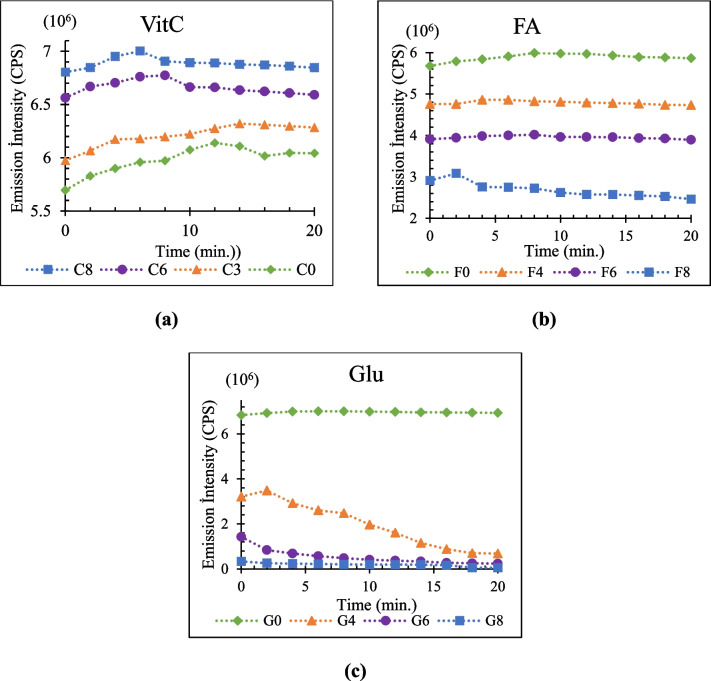
Incubation time graphics for (**a**) Vit C, (**b**) FA and (**c**) Glu

For incubation time optimization, four samples of each metabolite with varying concentrations (C_0_, F_0_ and G_0_ represent QD solution without metabolites, C3-1.7, C6-26.7 and C8-333 µg**/**mL containing Vit C, F4-3.5E-1, F6-3.5E + 1, F8-3.5E + 3 ng**/**mL containing FA and G4-0.25, G6-1, G8-4 mg**/**mL containing Glu) were prepared. For 20 min, the fluorescence measurements are taken at two-minute intervals, as Fig. 8 illustrates. The fluorescence responses of QDs stabilized at the ideal incubation time, which was determined to be 16 min for Vit C, 10 min for FA, and 16 min for Glu.

### Metabolite Detection Studies

**Fig. 9:**
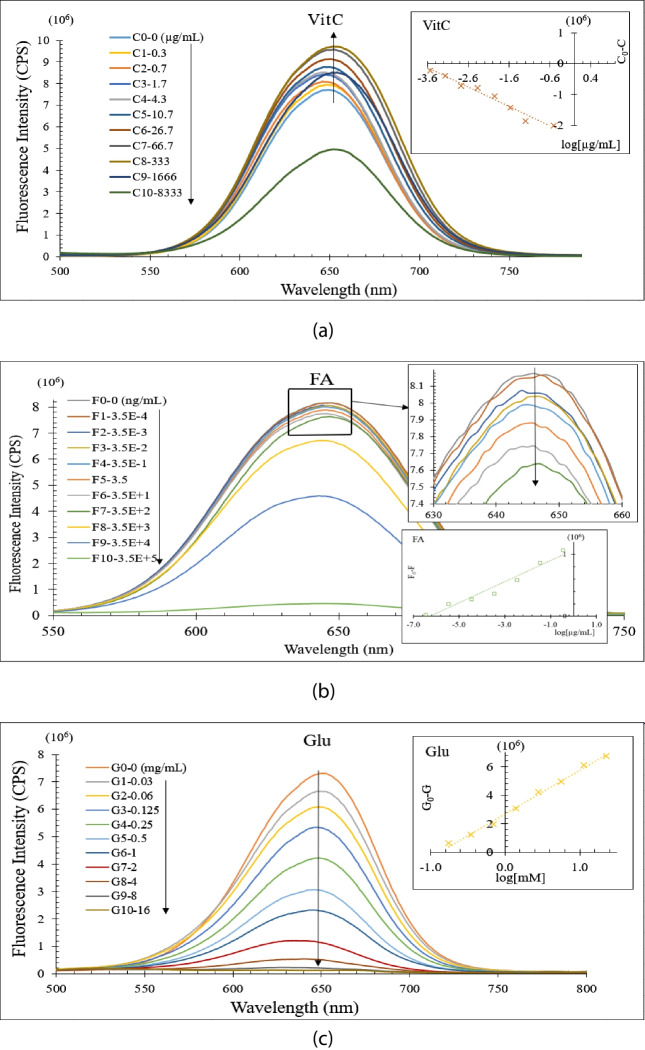
The fluorescence intensity responses and the linear relationship between I_0_-I and the logarithmic concentration of CdTe/ZnS QDs for (**a**) Vit C, (**b**) FA and (**c**) Glu

The fluorescence intensity responses of CdTe/ZnS QDs to changing metabolite concentrations (Vit C, FA and Glu) were obtained and given in Fig. 9 under the optimized conditions. The fluorescence intensities of FA and Glu decrease with increasing concentrations, whereas the fluorescence intensities of Vit C decrease with decreasing concentrations. Linear plots were produced by graphing against the logarithmic concentration of the metabolites.

Changes in fluorescence intensity of QDs in the presence of distinct metabolites can be attributed to particular chemical interactions. The observed reduction in fluorescence intensity with increasing quantities of FA and Glu is most likely due to quenching processes. In these instances, metabolites operate as quenchers, either statically or dynamically. Static quenching is the development of a non-fluorescent combination between QDs and metabolites in their ground state, preventing fluorescence emission [52]. Dynamic quenching, also known as collisional quenching, happens when quenchers contact with the excited state of QDs, resulting in a non-radiative deactivation of the excited state and a reduction in fluorescence intensity [53]. In contrast, for Vit C, the fluorescence intensity decreases with decreasing concentrations, which can be explained by its redox properties. Vitamin C is a strong reducing agent, and its redox interactions with QDs can alter their electronic states [54]. At higher concentrations, Vit C may reduce the QDs, changing their fluorescence characteristics. As the concentration of Vit C decreases, this reducing effect diminishes, resulting in a higher fluorescence intensity. The linear relationship observed by plotting I_0_ − I against the logarithmic concentration of the metabolites indicates that these interactions follow a predictable quenching mechanism, providing insights into the sensitivity and selectivity of QDs as fluorescent sensors for different metabolites [55]

In the Fig. 7, 0.3–1600 µg/mL, 3.5E^−6^–350 µg/mL and 0.35–90 mM linear ranges for Vit C, FA and Glu, high and acceptable R^2^ values as 0.9769, 0.9925 and 0.9919 were obtained, respectively. Therefore, there is high enough sensitivity in the suggested approach to identify these compounds in actual serum samples. Table 1 displays a comparison of R^2^ values and linear ranges between previous studies and it is seen that our technique gives strong R^2^ values and rather large linear range when compared to other works. To the best of the author's knowledge, CdTe/ZnS QDs with MPA ligands that are evaluated for the first time in the literature have competitive results that are in good agreement with the previous studies, according to this framework. The MPA based CdTe/ZnS QDs have been successfully demonstrated by these results to be a viable substitute for other QDs that have been extensively examined by earlier works.

The response of the CdTe/ZnS QDs sensor to FA and Glu is dependent on the selectivity of the sensor towards each metabolite. The study definitively shows that both metabolites cause fluorescence quenching through the Förster Resonance Energy Transfer (FRET) mechanism [37, 56]. However, the sensor's unique design and calibration allow it to discriminate between the two metabolites based on their distinct quenching profiles and linear ranges for detection. The response reflects the combined quenching effect, but the sensor can and does detect each metabolite separately due to its optimised selectivity. For example, in a real sample, CdTe/ZnS QDs showed different quenching for each metabolite, enabling the sensor to distinguish them even in a mixed solution by analysing the fluorescence decay attributed to the concentration of each analyte.

The claim for a redox reaction between VitC and CdTe/ZnS quantum dots is based on widely recognised principles in QDs and redox chemistry. VitC, known for its strong reducing properties, frequently exchanges electrons with materials capable of accepting electrons, such as metal-containing QDs. This interaction often results in quenching of fluorescence and this has been documented in several studies involving quantum dots and reducing agents [57].

### Fluorescence Sensing Mechanism

**Fig. 10:**
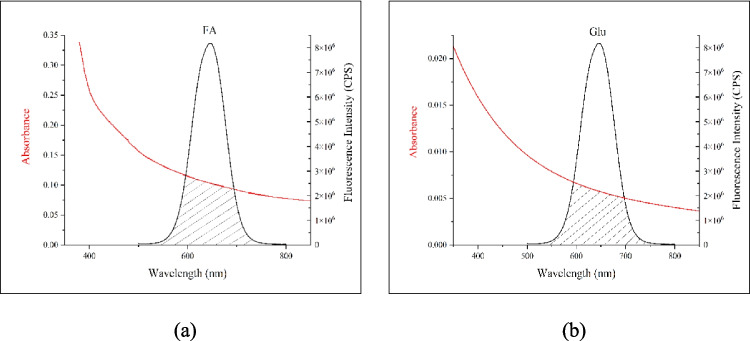
Spectral overlap between the emission spectrum of CdTe/ZnS QDs and the absorbance spectrum of (**a**) FA and (**b**) Glu

Fluorescence intensity changes of QDs is based on the process between QDs and metabolites interactions. Electron–hole pair recombination is the main source of the fluorescence of the QDs [58]. The interaction between the surfaces of QDs and metabolites affects the recombination between this electron–hole pair and causes a change in the fluorescence response [59]. Changes in fluorescence intensity can be explained by many kinds of physical mechanisms such as internal filter effect (IFE), electron transfer or nonradiative recombination [60, 61]. The values ​​obtained as a result of fluorescence measurements and calculations made with metabolites and QDs play an important role in understanding the interaction mechanism. Particularly in metabolite detection studies, biological intermolecular interactions with QDs are that the interaction occurs with intermolecular forces. Non-radiative and non-destructive energy transfer occurs depending on the distance between the donor (QD) and the acceptor (metabolite), with respect to the FRET mechanism [62, 63]. FRET involves energy transfer between QDs and quenching metabolites through dipole–dipole interactions, without the formation of a photon. The spectral overlap between the emission spectrum of the donor and the absorption spectra of the acceptors is the main case to explain the FRET mechanism and the overlap between FA and Glu by CdTe/ZnS QDs are shown in Fig. 10.

The efficiency of energy transfer (E) is determined by the distance between CdTe/ZnS QDs and metabolite, as stated in Eq. 2 [34],

2 $$E=\frac{{R}_{0}^{6}}{{R}_{0}^{6} + {r}_{0}^{6}}=1-\frac{F}{{F}_{0}}$$

where r_0_ is the distance between donor and acceptor, R_0_ is the Forster distance, F is the fluorescence intensity of QDs in the presence of metabolites and F_0_ is the fluorescence intensity in the absence of the metabolites [76]. By Eq. 3, the Forster distance, R_0_, is calculated,

3 $$\begin{array}{cc}{\text{R}}_{0}=0.211\times {\left({\text{k}}^{2}{\text{xn}}^{-4}\times\upphi \times \text{J}\left(\uplambda \right)\right)}^{\left(\frac{1}{6}\right)}& \left(A{\kern-3.5pt}^{^{\circ}}\right)\end{array}$$

with k^2^ is the orientation factor and generally accepted as 2/3, n is the refractive index of the medium and accepted as 1.33 for aqueous solution, ϕ is the PLQY which is obtained in the absence of the metabolites and calculated to be 28.22% for CdTe/ZnS QDs. Lastly, J is the overlap integral of QDs and metabolites and calculated by Eq. 4,

4 $$J={\int }\frac{F\left(\lambda \right)\epsilon \left(\lambda \right){\lambda }^{4}d\lambda }{F\left(\lambda \right)d\lambda }$$

where F(λ) is the fluorescence intensity of QDs, Ɛ(λ) is the molar extinction coefficient of metabolites [80].

While R_0_, r_0_ and r_0_/R_0_ values for FA and Glu are given in Table 3, the fluorescence enhancing effect of Vit C on QD is not explained by the FRET mechanism and is not given in this table For explanation of FRET mechanism occurred between QDs and metabolites, while R_0_ should be in the range of 20–90 Å and r_0_ should be in the range of 10–100 Å [34, 69], [81]. Besides, the energy is transferred efficiently between acceptor and donor when r_0_/R_0_ remains in the range of 0.5–2 [34]. Therefore, the quenching mechanism can be attributed to FRET type energy transfer between FA, and Glu and the excited state of CdTe QDs.

**Table 3:** Forster distances (R_0_) and distance between QDs and metabolites (r_0_) and their ratio for FA and Glu

	R_0_ (Å)	r_0_ (Å)	r_0_/R_0_
FA	75.88	97.82	1.29
Glu	79.88	40.86	0.51

The ratio r₀/R₀ for folic acid is 1.29, which is within the range of 0.5–2 where energy transfer can take place efficiently. This suggests that folic acid can interact with CdTe/ZnS QDs via an effective FRET mechanism. However, the r₀ value (97.82 Å) is slightly higher than the R₀ value (75.88 Å). This means that the energy transfer is not exactly at the most efficient distance, but still indicates that an effective FRET mechanism can still be realised.

The ratio r₀/R₀ for glu is 0.51, which is within the range of 0.5–2 where energy transfer can occur efficiently. This indicates that glucose can interact with CdTe/ZnS QDs via an efficient FRET mechanism. The r₀ value (40.86 Å) is considerably smaller than the R₀ value (79.88 Å). This means that energy transfer can take place very efficiently. The small distance between glucose and QDs makes the energy transfer more efficient. The possibility of an efficient FRET mechanism between FA and CdTe/ZnS QDs is high, but the efficiency of energy transfer may be slightly lower than the interaction with glucose, given the distances. The energy transfer efficiency of the interaction between Glu (glucose) and CdTe/ZnS QDs is highly energetic and these findings strengthen the potential of CdTe/ZnS QDs for use in biomedical applications such as biosensors, imaging agents and drug delivery systems. In particular, the high energy transfer efficiency of glucose suggests that this system can be used in the monitoring of glucose levels.

The redox reactions between Vit C and QDs can affect their fluorescence through a variety of mechanisms, mainly involving electron transfer processes. These such interactions can enhance or quench the fluorescence of QDs depending on the properties of the system, including the type of QDs, surface modifications, and the presence of other molecules or ions in the environment [64]. Figure 7(a) shows how adding Vit C to CdTe/ZnS QDs caused the FL intensity to progressively rise until 333 µg/mL. The increased FL intensity difference (C_0_-C) of the CdTe/ZnS QDs and the logarithm of the additional Vit C concentration have a good linear relationship, as seen in the inset to Fig. 7(a). The fact that Vit C causes an increase in FL intensity in CdTe/ZnS QDs is attributed to the defects on the surface of the QDs [65]. While the QDs are synthesized, since the atoms on their surfaces cannot bond effectively with the ligands on the QD walls, defects form on the surfaces of the QDs, causing excited electrons to pass into valence bands without radiation. In this study, it is thought that the increase in FL intensity with the increase in Vit C concentration occurs because Vit C has a healing effect on the defects of the QDs and prevents the non-radiative transition of electrons. Similar trends and conclusions were also reported in studies on the PL-enhancing effect of Vit C [63, 66] Additionally, Vit C can serve as surface caps by reducing the atoms and MPA molecules on the surface of QD to eliminate surface defects [67]. With the addition of C9 and C10 to QDs, the density of Vit C in the solution increased significantly and became higher than the surface defects. It is thought that due to this excess, the distance between Vit C and QD decreases and the FRET mechanism becomes active, thus causing the fluorescence of QDs to be quenched.

### Interference Study

**Fig. 11:**
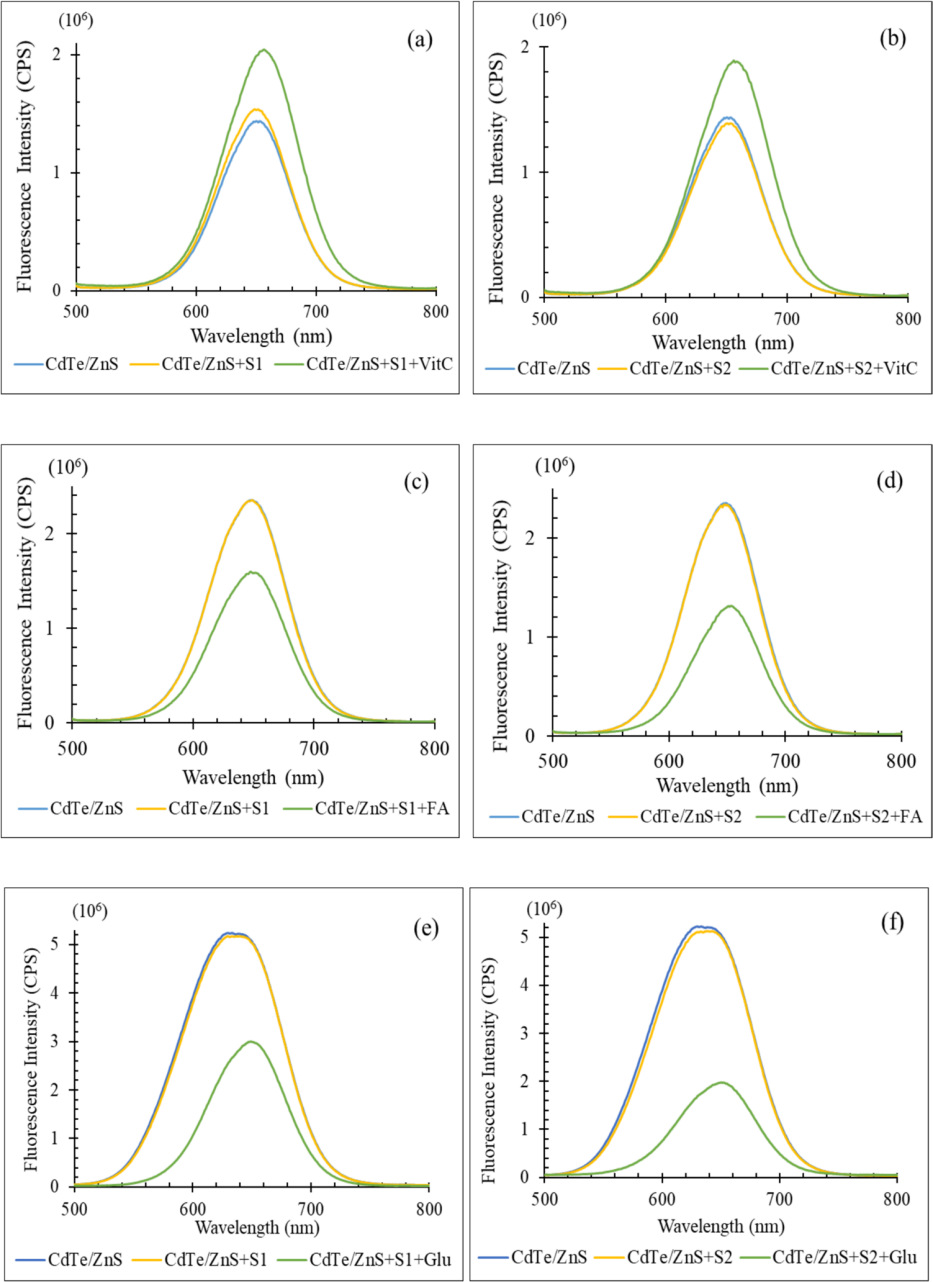
Selectivity studies of CdTe/ZnS QDs in heavy metal ions (S1) and metal ions (S2) for (**a**), (**b**) Vit C, (**c**), (**d**) FA and (**e**), (**f**) Glu

As shown in Fig. 11, selectivity studies were conducted by CdTe/ZnS QDs against interference substances while detection of Vit C, FA and Glu. The complex matrices like blood have so many substances that affect the fluorescence intensity of QDs. Because of this, for discrimination of the effects of them on FL intensity of QDs, selectivity studies were completed with two different solutions. While one of them (S1) includes heavy metal ions like Pb^+2^, Sb^+3^, Hg^+1^, As^+3^, Cd^+2^, another (S2) consists of metal ions like Ca^+2^, K^+1^, Na^+1^, Mg^+2^, Fe^+2^, Cu^+2^, Co^+2^, Zn^+2^, Mn^+2^, Ni^+2^, Mo^+2^, B^+3^ included in the composition of blood. In Fig. 9, CdTe/ZnS solution indicates blank solution which has no metabolites or interference substances. CdTe/ZnS + S1/S2 show the solution that added just interference substances to QDs solution and it is used to display the effect of the interferences on FL intensity of QDs. Lastly, CdTe/ZnS + S1/S2 + Metabolite solutions are used for impacts of addition of metabolites over the complex matrices including QDs solutions. While adding interference solutions to QD solution, the FL intensity of QDs slightly change as 7.06% in Fig. 9(a), 3.12% in Fig. 9(b), 0.20% in Fig. 9(c), 0.58% in Fig. 9(d), 1.04% in Fig. 9(e) and 1.84% in Fig. 9(f). However, this percent and FL intensity changed considerably after adding metabolites to CdTe/ZnS + S1/S2 solutions as 42.17% in Fig. 9(a), 31.46% in Fig. 9(b), 32.06% in Fig. 9(c), 44.04% in Fig. 9(d), 42.74% in Fig. 9(e) and 62.22% in Fig. 9(f). This proves that the change in FL intensity of QDs is caused by metabolites and the selectivity of this detection system against metal ions and metabolites is excellent.

### Real Sample Analysis

To further applicability of the developed fluorescence sensor, amounts of Vit C, FA and Glu were detected in 3 different human blood serum samples by using standard addition method. In this method, added amounts are determined close to the normal level of metabolites in the blood sample in order to minimize the matrix effect of complex solutions like blood. In real sample analysis, known amounts of metabolites were added to blood serum samples and then detection of these amounts were successfully accomplished similar to previous studies [36, 67]. The added and found amounts of metabolites and calculated error are shown in Table 4. While linear detection ranges are found to be 10–20 µg/mL for Vit C, 7.5–20 ng/mL for FA and 0.25–1.5 mg/mL for Glu, the recoveries are in the range of 96.31–104.36% for Vit C, 95.6–103.94% for FA and 95.33–104.67% for Glu with the error no more than 5% in three blood serum samples. These findings clearly demonstrate the potential applications of the MPA-CdTe/Zns QD-based detection system developed for this study in the quantification of metabolites in the real samples. As a result, it is clearly seen that CdTe/ZnS QDs synthesized with MPA ligand can compete with existing QDs synthesized with different ligands preferred in the detection of Vit C, FA and Glu and can be used instead of them.

**Table 4:** Determination of Vit C, FA and Glu in 3 real blood serum samples

		Blood Sample 1		Blood Sample 2		Blood Sample 3	
	Added	Found	Error (%)	Found	Error (%)	Found	Error (%)
**Vit C** **(µg/mL)**	10	9.654	3.46	10.372	3.72	9.733	2.67
	12.5	12.331	1.35	12.242	2.06	13.044	4.36
	15	14.799	1.34	14.447	3.69	14.636	2.43
	17.5	17.700	1.14	16.995	2.89	16.961	3.08
	20	19.882	0.59	19.450	2.75	20.942	4.71
**FA** **(ng/ml)**	7.5	7.232	3.57	7.769	3.59	7.617	1.55
	10	10.145	1.45	10.124	1.24	10.245	2.45
	12.5	12.135	2.92	11.950	4.40	12.993	3.94
	15	15.548	3.65	14.493	3.38	14.439	3.74
	17.5	17.587	0.50	17.315	1.06	17.47	0.18
	20	19.512	2.44	20.789	3.94	19.967	0.16
**Glu** **(mg/mL)**	0.25	0.239	4.46	0.243	2.64	0.247	1.32
	0.5	0.485	2.99	0.523	4.67	0.514	2.79
	0.75	0.778	3.71	0.744	0.75	0.783	4.39
	1	0.999	0.08	0.966	3.37	0.996	0.40
	1.25	1.192	4.67	1.201	3.92	1.276	2.10
	1.5	1.452	3.19	1.458	2.82	1.514	0.94

Table 5 compares the performance of various QDs used in the detection of metabolites such. The parameters compared include the linear detection range and the limit of detection (LOD), as reported in previous studies. The results obtained in this study are highlighted and compared to those in the literature. For FA detection, the linear range achieved in this study using CdTe/ZnS QDs was 0.0198–1.980 µM, with a LOD of 0.476 µM. Compared to other reported studies, such as MoS2 QDs (LOD: 0.1 µM, range: 0.1–125 µM) [68] and CdSeTe QDs (LOD: 0.84 µg/mL, range: 3.5E-6–350 µg/mL) [40], our results demonstrate a competitive detection limit within a narrower yet analytically significant range. Moreover, compared to CQDs (LOD: 0.079 µM, range: 0.5–200 µM) [69], the obtained LOD is slightly higher; however, the material's stability and reproducibility in this study provide significant advantages. In the case of VitC detection, the CdTe/ZnS QDs in this study achieved a detection range of 1.7–9,070 µM and an LOD of 0.653 µM. When compared to other systems, such as Fe-CQDs (LOD: 0.133 µM) [70] or N-CQDs (LOD: 0.05 µM) [71], the LOD is slightly higher. However, the broader detection range observed in this study (spanning millimolar concentrations) underscores its suitability for applications requiring high dynamic range measurements. For Glu detection, the CdTe/ZnS QDs exhibited a linear range of 350–90,000 µM, with an LOD of 330 µM. This is comparable to other reported systems, such as CdTe/CdS QDs (LOD: 0.05 mM, range: 0.1–5 mM) [72] and CsPbBr3@SiO2 QDs (LOD: 18.5 µM, range: 20–400 µM) [73]. While the LOD is slightly higher, the extended linear range makes it highly advantageous for applications involving high-concentration glucose measurements.

**Table 5:** Comparison table of the study with the literature

QDs	Metabolites	Linear Range	LOD	Ref
MoS2	FA	0.1–125 μM	0.1 μmol/L	[68]
CdTe/ZnS	FA	0.1–50 μM	0.2 μM	[37]
CdSeTe	FA	3.5E^−6^–350 μg/mL	0.84 μg/mL	[40]
CQD	FA	0.5–200 µM	0.079 µM	[69]
CdTeS- QDs@SiO2	FA	5–80 µM	0.3 µM	[74]
CdTe/ZnS	FA	0.0198–1.980 µM	0,476 µM	This study
CdSe/ZnS	VitC	2–100 mg/L	0.6 mg/L	[75]
CdTe	VitC	0.1–1 μM	6.6 nM	[34]
CdSeTe	VitC	0.3–1600	1.15 μg/mL	[40]
Fe-CQDs	VitC	3.3–32.2 (µM)	0.133 (µM)	[70]
N-CQD	VitC	0.3–11.5 µmol/L	0.05 µmol/L	[71]
CdTe/ZnS	VitC	1.7–9,070 µM	0,653 µM	This study
CdSeTe	Glu	0.35–90 mM	0.33 mM	[40]
CdTe/CdS	Glu	0.1–5 mM	0.05 mM	[72]
CsPbBr3@SiO2	Glu	20–400 μM	18.5 μM	[73]
CQD	Glu	25–250 μM	2.2 μM	[76]
CdTe/ZnS	Glu	350–90,000 µM	330 µM	This study

The results of this study highlight the versatility and efficacy of CdTe/ZnS QDs for metabolite detection. While certain systems, such as carbon dots or Fe-CQDs, achieve lower detection limits, the broader linear ranges and consistent stability observed in this study provide significant advantages for practical applications. Additionally, the methodology utilized in this study offers a reproducible and scalable approach for developing QD-based sensors

## Conclusion

The development of CdTe/ZnS quantum dot-based fluorescence sensors, as explored in this study, represents a significant advancement in the detection of critical metabolites, including folic acid, glucose, and vitamin C. The successful application of QDs in biological sensing reaffirms their potential in diagnostic technologies due to their unique optical properties, including tunable fluorescence, size-dependent emission, and high quantum yield. This study demonstrates that QDs, specifically those comprising a CdTe/ZnS core/shell structure, are capable of offering sensitive, selective, and reliable detection mechanisms for metabolites in real biological matrices, such as blood serum, through a FRET quenching mechanism.

The findings demonstrate that under optimized conditions, the detection limits achieved for folic acid (0.84 µg/mL), glucose (0.33 mM), and vitamin C (1.15 µg/mL) are comparable, if not superior, to those reported in previous studies involving other QD systems, such as CdSeTe or carbon-based QDs. These findings underscore the suitability of CdTe/ZnS QDs for practical biomedical applications where both sensitivity and selectivity are paramount. The utilization of a FRET-based quenching mechanism serves to reinforce the dependability of this detection method, thereby providing a robust foundation for prospective advancements in metabolite sensing technologies.

Quantum dots (QDs) such as CdSe/ZnS have been shown to offer distinct advantages in metabolite detection when compared with conventional methods, including FITC labelling and antibody-conjugated fluorescent markers. These advantages encompass higher photostability, resistance to photobleaching, tunable wavelengths and the capacity to excite multiple emission bands with a single laser. A range of studies have been conducted to compare CdSe/ZnS QDs with other materials such as InP/ZnS, CuInS2/ZnS and carbon-based nanodots (NCDs). These studies highlight efforts to identify safer materials with similar optical benefits. While other methods, such as gold nanocrystal modified electrodes, also show promise, QDs stand out due to their versatility in bioconjugation, photostability and potential for advanced detection applications. One of the most noteworthy aspects of this study is the biocompatibility and aqueous solubility of the synthesized QDs, which have been facilitated by the mercaptopropionic acid (MPA) ligand. These factors not only enhance the sensor's practical application in biological systems but also align with the growing trend towards eco-friendly and non-toxic nanomaterials in biomedical technologies. The versatility of the CdTe/ZnS QDs demonstrated here also opens pathways for their integration into multiplex detection systems, where the simultaneous detection of multiple analytes could greatly improve diagnostic accuracy and efficacy.

## Data Availability

No datasets were generated or analysed during the current study.

## Declarations

Every experiment was conducted in compliance with relevant laws and institutional rules, and signed informed consent was obtained from human subjects for all samples. It was conducted within the framework of ethical permissions by the Düzce University Non-Interventional Health Research Ethics Committee.
